# Efficacy of orthokeratology lens with the modified small treatment zone on myopia progression and visual quality: a randomized clinical trial

**DOI:** 10.1186/s40662-024-00403-3

**Published:** 2024-09-02

**Authors:** Ganyu Gong, Bi Ning Zhang, Tengyou Guo, Guoying Liu, Ju Zhang, Xiu Juan Zhang, Xianli Du

**Affiliations:** 1 Eye Institute of Shandong First Medical University, Qingdao Eye Hospital of Shandong First Medical University, 5 Yanerdao Road, Qingdao, Shandong, 266071 China; 2 State Key Laboratory Cultivation Base, Shandong Provincial Key Laboratory of Ophthalmology, Qingdao, Shandong, China; 3 School of Ophthalmology, Shandong First Medical University, Jinan, Shandong China; 4 Shandong First Medical, University& Shandong Academy of Medical Sciences, Jinan, Shandong China; 5 Eye Institute of Shandong First Medical University, Eye Hospital of Shandong First Medical University (Shandong Eye Hospital), Qingdao, Shandong, China; 6 Department of Ophthalmology and Visual Sciences, The Chinese University of Hong Kong, Hong Kong, China; 7 Joint Shantou International Eye Center of Shantou University and the Chinese University of Hong Kong, Shantou, Guangdong, China

**Keywords:** Orthokeratology lens, Small treatment zone, Axial elongation, Objective visual quality

## Abstract

**Background:**

To evaluate the long-term effectiveness of orthokeratology (ortho-K) lenses with small treatment zone (STZ) or conventional treatment zone (CTZ) in controlling axial elongation in children with myopia as well as the impact on visual quality. We also sought to determine the effect of retinal visual signal quality on axial elongation.

**Methods:**

This is a prospective randomized controlled study. A total of 140 participants (age ranging from 8 to 12 years) were randomly assigned to wear either STZ or CTZ ortho-K lenses. STZ ortho-K lenses design was achieved by changing the depth of reverse zone and the sagitta height of the optical zone. Using the IOL-Master 500, axial length (AL) was measured at baseline and after 6, 12 and 18 months of ortho-K treatment. Spherical aberration (SA) and corneal topographic parameters were obtained by the Pentacam anterior segment analyzer at baseline and the 1-month follow-up visit, and optical qualities were assessed by optical quality analysis system-II (OQAS-II) at baseline and after 1 month of lens wearing. Optical quality parameters mainly included the modulation transfer function (MTF) cutoff, Strehl ratio (SR), objective scattering index (OSI), and predicted visual acuity (PVA).

**Results:**

A total of 131 participants completed the study, including 68 in the STZ group and 63 in the CTZ group. The STZ group had significantly reduced AL elongation compared to the CTZ group after treatment (12 months: 0.07 ± 0.11 mm vs. 0.14 ± 0.12 mm,* P* = 0.002; 18 months: 0.17 ± 0.15 mm vs. 0.26 ± 0.16 mm, *P* = 0.002). The topography in the STZ group showed a smaller treatment zone (TZ) diameter (2.50 ± 0.23 mm vs*.* 2.77 ± 0.18 mm, *P* < 0.001), a wider defocus ring width (2.45 ± 0.28 mm vs. 2.30 ± 0.30 mm, *P* = 0.006), and larger values of total amount of defocus (119.38 ± 63.71 D·mm^2^ vs. 91.40 ± 40.83 D·mm^2^, *P* = 0.003) and total SA (0.37 ± 0.25 μm vs*.* 0.25 ± 0.29 μm, *P* = 0.015), compared with the CTZ group. Objective visual quality decreased in both groups (*P* < 0.001). This was evidenced by a greater decrease in MTF cutoff (− 14.24 ± 10.48 vs. − 10.74 ± 9.46, *P* = 0.047) and SR values (− 0.09 ± 0.07 vs. − 0.06 ± 0.07, *P* = 0.026), and an increase in OSI value (0.84 ± 0.72 vs. 0.58 ± 0.53, *P* = 0.019). PVA9% decreased significantly in the STZ group but not the CTZ group. A statistically significant negative correlation was found between the changes in total SA and MTF cutoff values (*r* =  − 0.202, *P* = 0.025). AL changes were associated with sex, change of MTF cutoff value, increment of total SA and TZ area.

**Conclusions:**

Compared with CTZ ortho-K lenses, STZ ortho-K lenses significantly inhibited axial elongation in children with myopia while moderately reducing their objective visual quality. Axial elongation was affected by retinal visual quality, and it may be a possible mechanism for ortho-K slowing myopia progression.

*Trial registration* This trial is registered at Chinese Clinical Trial Registry on November 5, 2019 with trial registration number: ChiCTR1900027218. https://www.chictr.org.cn/showproj.html?proj=45380

**Supplementary Information:**

The online version contains supplementary material available at 10.1186/s40662-024-00403-3.

## Background

The rising global prevalence of myopia is a significant concern, with projections indicating that by 2050, approximately half of the world’s population will be suffering from myopia, with one in ten being highly myopic [1]. Pathological myopia is associated with severe ocular complications due to the excessive increase in axial length (AL) [2]. Various strategies have been developed for myopia control, including increasing outdoor time, utilizing pharmaceutical approaches such as low-dose atropine, and employing optical treatments [3–5].

Orthokeratology (ortho-K) has gained considerable popularity in the last decade, with a myopia control efficacy of about 30% to 60% [6–9]. Throughout ortho-K treatment, a notable flattening of the central cornea occurs, resulting in a significant improvement in daytime uncorrected visual acuity (UCVA) [10]. This flattened region is referred to as the treatment zone (TZ). Concurrently, the mid-peripheral cornea steepens, creating a myopic defocus on the peripheral retina when light passes through this area. This myopic defocus aids in controlling the progression of myopia [11]. On the other hand, while ortho-K lenses improve UCVA and slow axial elongation, they may also lead to corneal irregularity, resulting in increased higher-order aberration (HOA) such as spherical aberration (SA) and reduced visual quality [12–17].

Different designs of ortho-K lenses have varying effectiveness in slowing the progression of myopia. Currently, there are several randomized trials evaluating the efficacy of different designs of ortho-K lenses for myopia control. A retrospective study showed that ortho-K lenses with a relatively small TZ size and significant decentration on the corneal surface can help slow myopia progression in children [18]. A randomized clinical trial revealed that ortho-K lenses with smaller back optical zone diameters (BOZD) resulted in smaller TZ diameters, leading to a delay in axial elongation over 2 years [19]. Moreover, only a limited number of studies have examined the effect of visual quality following ortho-K treatment. Few studies have examined both myopic control efficacy and corresponding changes in visual quality with different designs of ortho-K lenses.

We conducted a prospective randomized controlled trial with an 18-month follow-up to investigate the efficacy of different designs of ortho-K lenses on myopia control and their impact on visual quality. Interestingly, this study also analyzed the effect of retinal visual signals on myopia control. Our findings indicate that effective myopia control is achieved at the expense of visual quality. This provides new insights into the clinical use of ortho-K for myopia control.

## Methods

This study was a randomized, double-masked trial conducted at Qingdao Eye Hospital of Shandong First Medical University. Recruitment began in January 2021 and was facilitated through online advertising and referrals from existing participants. Randomization was conducted by an independent clinical research coordinator using a table generated by the SAS program (SAS Institute, Cary, NC, USA). Eligible children were then randomly allocated to either the small treatment zone (STZ) group or the conventional treatment zone (CTZ) group based on their order of presentation at the hospital. Follow-up clinical examinations were carried out by another clinical research coordinator, who was blinded to the participants’ subgroups. The study adhered to the tenets of the Declaration of Helsinki and received approval from the Ethics Committee of Qingdao Eye Hospital (2019-28). This trial was registered with the Chinese Clinical Trial Registry (ChiCTR1900027218). Verbal consent was obtained from participating children, and written informed consent was obtained from their parents or guardians.

### Trial participants

Participants were randomly assigned in a 1:1 ratio to wear either the modified three-zone STZ design (STZ group) or the conventional four-zone design (CTZ group) ortho-K lenses for both eyes. The inclusion criteria were as follows: age 8–12 years, spherical refraction between − 4.00 to − 0.75 diopter (D), cylinder refraction ≤ 1.25 D, best-corrected logarithm of the minimum angle of resolution (logMAR) visual acuity of 0.10 or better in both eyes, absence of ocular diseases, ocular trauma, or previous ocular surgery, no contraindications to corneal contact lenses, and no history of contact lens wear. Exclusion criteria included a history of myopia control treatment, strabismus or amblyopia, ocular inflammation or infection, corneal dystrophy, and poor compliance with lens wear.

### Lens design and fitting

The STZ ortho-K lens is a modification of the original three-zone design to achieve a smaller TZ, intended to improve myopia control while maintaining good tolerance to corneal irregularity [20]. The STZ ortho-K lens was manufactured using HDS 100 material (CRT; Paragon Vision Sciences, USA). This design was achieved by elevating the depth of the reverse zone and adjusting the sagitta height of the optical zone to result in a smaller TZ (Figure S1, Table S1). As for the specific quantitative change of reverse zone depth (RZD), it needs to be customized according to the fitness evaluation of the lens worn by each child. In contrast, the CTZ lens used a conventional four-zone reverse geometry design (Alpha Corp., Japan). Although both ortho-K lenses have the same BOZD (6.0 mm), different designs of other parameters produced different TZ sizes on the anterior corneal surface after ortho-K treatment. Detailed lens parameters are described in Table S1. Lens fit was assessed using fluorescein staining under cobalt blue light in a slit lamp microscope, and necessary adjustments were made to the lens parameters as needed. Participants were instructed to wear ortho-K lenses every night for a minimum of eight consecutive hours throughout the study. Follow-up visits were scheduled at 1 day, 1 week, 1 month, 3 months, 6 months, 9 months, 12 months, and 18 months after lens wear.

### Measurement

Comprehensive examinations were conducted on the children at baseline and during each follow-up visit. These examinations included cycloplegic refraction, UCVA, intraocular pressure (IOP) measurement, slit-lamp examination, and corneal topography using the Pentacam anterior segment analysis system (Oculus, Germany). Cycloplegic refraction was conducted by administering 0.5% compound tropicamide eye drops, with four drops given at 5-min intervals followed by a 20-min wait after the last drop until cycloplegia was completely achieved. Refraction was measured using an autorefractor (KR-8900, Topcon Corp., Japan) and the average of three automatic measurements was analyzed. Subjective refraction was performed by the same optometrist. The spherical equivalent refraction (SER) was calculated as the spherical power plus 1/2 the cylindrical power. Visual acuity was assessed using the logMAR chart under normal lighting before cycloplegia. Data on AL and pupil diameter (PD) were obtained by the IOL-Master 500 (ZEISS Corp., Germany) at baseline and every follow-up visit. Objective visual quality was measured using the optical quality analysis system-II (OQAS-II, Visiometrics Corp., Spain), a widely used tool with proven repeatability for assessing optical quality [21–23]. Measurements were taken before and after 1-month lens wear. To ensure the accuracy of the collected data, children were instructed to adapt to a dark environment for 10 min prior to examination to ensure a natural pupil size of at least 4 mm. Before the examination, the child’s refractive error was corrected using the OQAS in-built compensating lens (up to 4.00 D) or an insert lens correction (greater than 4.00 D).

### Topographic maps parameters

**Fig. 1:**
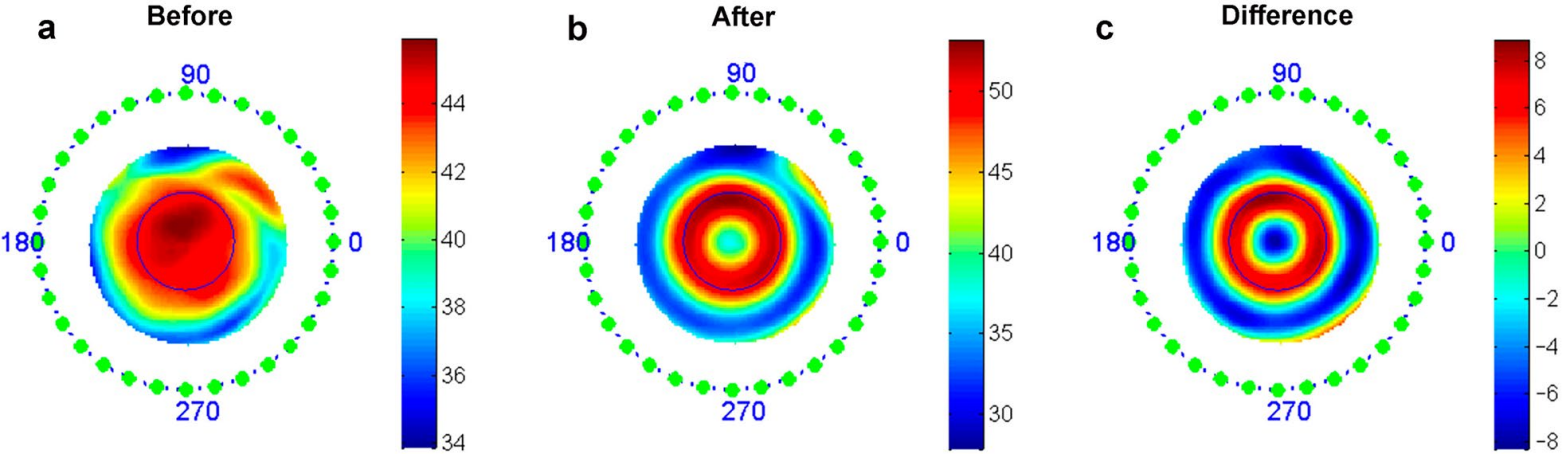
MATLAB software was used to analyze differences in topographic maps of myopic children at different time points to obtain topographic parameters. **a** Tangential curvature map at baseline; **b** Tangential curvature map at 1-month visit; **c** The difference map used to determine the treatment zone (TZ) parameters (TZ size, area, decentration distance, etc.)

Examinations of corneal topography were taken by the Pentacam instrument during the follow-up visits. We collected total SA and HOA values before and after 1 month of treatment. A specially written MATLAB software was used to analyze the tangential subtractive maps between baseline and 1-month follow-up visits (Fig. 1). The quadratic fitting method was used to map and calculate the optical correction region, yielding data on the TZ diameter and area, eccentric distance of lenses, and defocus ring width. We used a PD value of 4.70 mm (measured by the IOL Master-500) to limit the calculation range in the MATLAB software. This approach allowed us to determine the effective width of the defocus ring and the effective total amount of defocus (the total value of defocus · area) within PD range. These indicators described the corneal morphology produced on the anterior corneal surface by the different designs of ortho-K lenses.

### OQAS-II parameters

To assess the impact of diffraction and scattering on visual quality, we employed the dual-channel OQAS-II system. The OQAS-II captures images of point light sources on the retina using a dual-channel technique, enabling the analysis of the point spread function (PSF), which could be transformed into a modulation transfer function (MTF) after Fourier conversion. Then, various visual quality parameters, including the MTF cutoff frequency, Strehl ratio (SR), objective scatter index (OSI) and predicted visual acuity (PVA) were obtained [24].

The MTF is a function used to describe the transmission performance of refractive systems by measuring the relative amplitude of the modulation degree of sinusoidal stripes with different spatial frequencies after passing through the optical system ranging from 0 to 1. The MTF cutoff value indicates the image quality of the ocular refractive system – the higher the MTF cutoff value, the better the image quality. The SR values represent the ratio of the central illumination point of a real eye with aberration to that of an ideal eye without aberration, assuming the same PD. The OSI is defined as the ratio of light in the peripheral annular region to the light in the central peak of the acquired system. The PVA is derived from the MTF curve, representing the contrast visual acuity under three contrast conditions: 100% (PVA100%), 20% (PVA20%), and 9% (PVA9%). These values correspond to the optical acuity (logMAR) during daytime, dusk, and nighttime, respectively.

### Subjective visual quality questionnaire

The subjective visual quality questionnaire is based on the National Eye Institute Refractive Error Quality of Life Instrument (NEI-RQL-43) [25–27]. The modified questionnaire is more applicable to children wearing ortho-K lenses. Administered concurrently with objective visual quality measurements, it reflects changes in children's subjective visual quality.

### Sample size calculations

To determine the required number of study subjects, a minimum sample size of 22 per group was calculated, ensuring a power of 0.90 to detect a change of 0.16 mm (SD 0.10 mm) in AL elongation between the two groups over 12 months with an α-value of 0.05, based on our previous published study [28]. A total of at least 27 cases per group were targeted for recruitment, taking into account a projected dropout rate of 20%, resulting in a total sample size of at least 54 cases across the two groups.

### Statistical analysis

Statistical analysis was performed using SPSS version 26.0 (IBM, ibm.com). Analysis included data only from the right eye of each subject. The Shapiro–Wilk’s test was used to test the normality of the data. For normally distributed data, descriptive statistics are expressed as mean ± standard deviation, and unpaired t-tests were applied in baseline data comparison. The multivariate repeated measures analysis of covariance (ANCOVA) was applied for comparison of the AL changes between the two groups to control the influence of confounding factors such as age, sex and diopter. For data with non-normal distribution, the Mann–Whitney test was performed. Factors that may affect 18-month AL changes were examined using Pearson correlation analysis. Univariate and multivariate regression analyses were also constructed to explore the association between AL elongation and ocular parameters. Two-tailed tests were employed, and a significance level of 0.05 was set. The internal repeatability of measurements was assessed using the intraclass correlation coefficient (ICC) calculated through reliability analysis in SPSS, employing a two-way random model with absolute agreement.

## Results

### Demographics and ocular biometrics of study participants at baseline

**Fig. 2:**
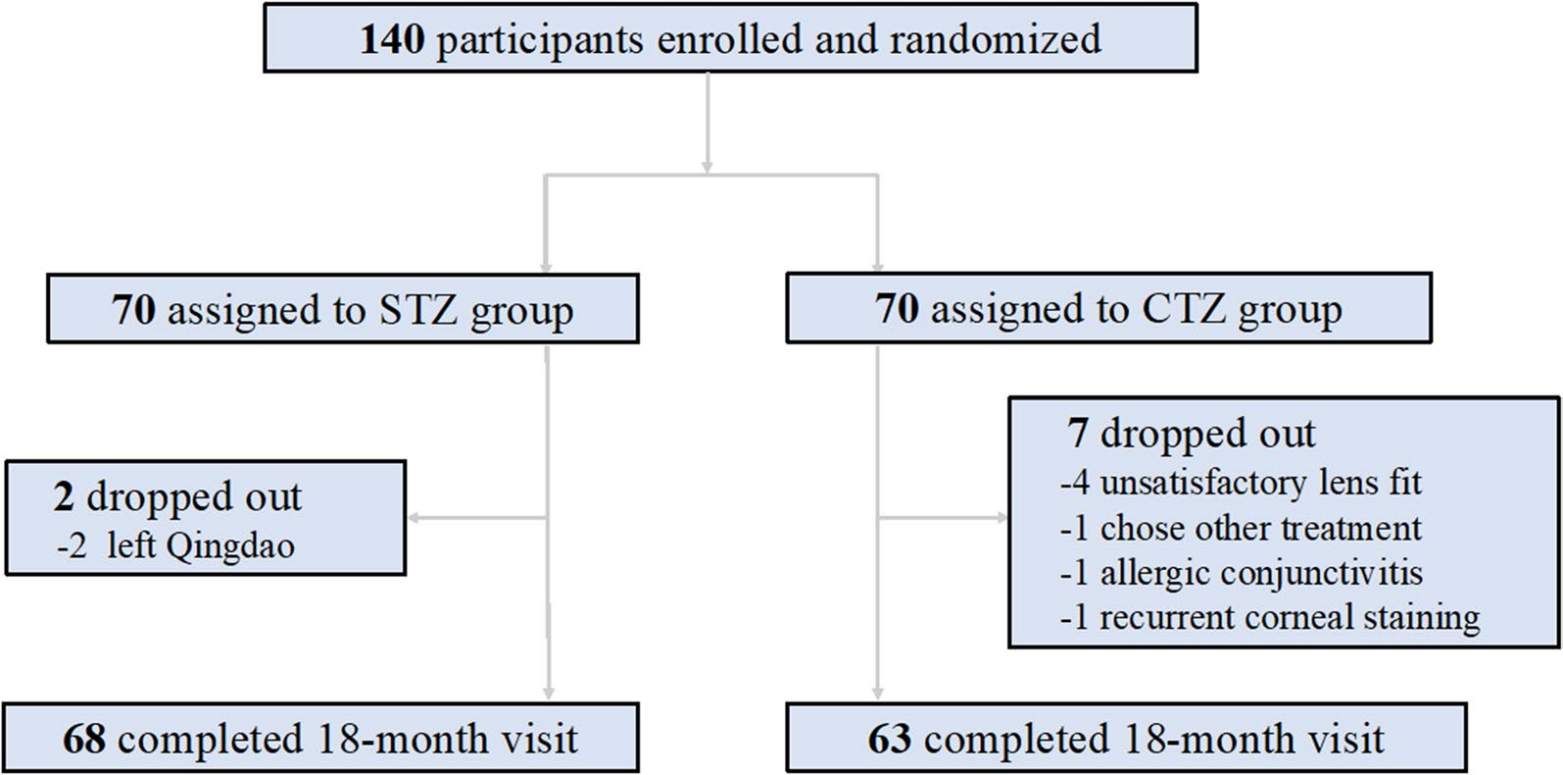
Study flow diagram. STZ, small treatment zone; CTZ, conventional treatment zone

A total of 140 myopic children who met the recruitment criteria were initially enrolled in the study and randomly assigned to either the STZ or the CTZ group. They were followed up for 18 months. However, during the study, 2 subjects from the STZ group and 7 subjects from the CTZ group failed to attend follow-up visits, resulting in their exclusion from the study (Fig. 2). Therefore, the final analysis included 131 children: 45 males and 98 females. Among them, 68 children were in the STZ group and 63 were in the CTZ group. At baseline, there were no statistically significant differences between the two groups in age, sex, SER, UCVA, PD and AL (all* P* > 0.05, Table 1).

**Table 1:** Demographics and ocular biometrics of myopic children at baseline

Variable (mean ± SD)	STZ group (n = 68)	CTZ group (n = 63)	t value	*P* value
Age (years)	9.94 ± 1.52	10.17 ± 1.88	− 0.765	0.446
Sex (M:F)	20:48	25:38	1.231	0.221
Sphere (D)	− 1.92 ± 0.85	− 2.07 ± 0.92	0.976	0.331
Astigmatism (D)	− 0.47 ± 0.53	− 0.43 ± 0.41	− 0.484	0.629
SER (D)	− 2.15 ± 0.93	− 2.28 ± 0.93	0.805	0.422
UCVA (logMAR)	0.77 ± 0.31	0.72 ± 0.56	0.611	0.542
PD (mm)	4.71 ± 0.98	4.70 ± 1.06	0.083	0.934
AL (mm)	24.39 ± 0.61	24.61 ± 0.76	− 1.774	0.079

Normally distributed data were expressed as mean ± standard deviation

*Sphere =* spherical refraction; *SER =* spherical equivalent refraction; *UCVA =* uncorrected visual acuity; *PD =* pupil diameter; *AL =* axial length; *n =* number of subjects

^*^P < 0.05 indicates statistical significance

### Changes in AL, SER, and UCVA after ortho-K treatment

**Fig. 3:**
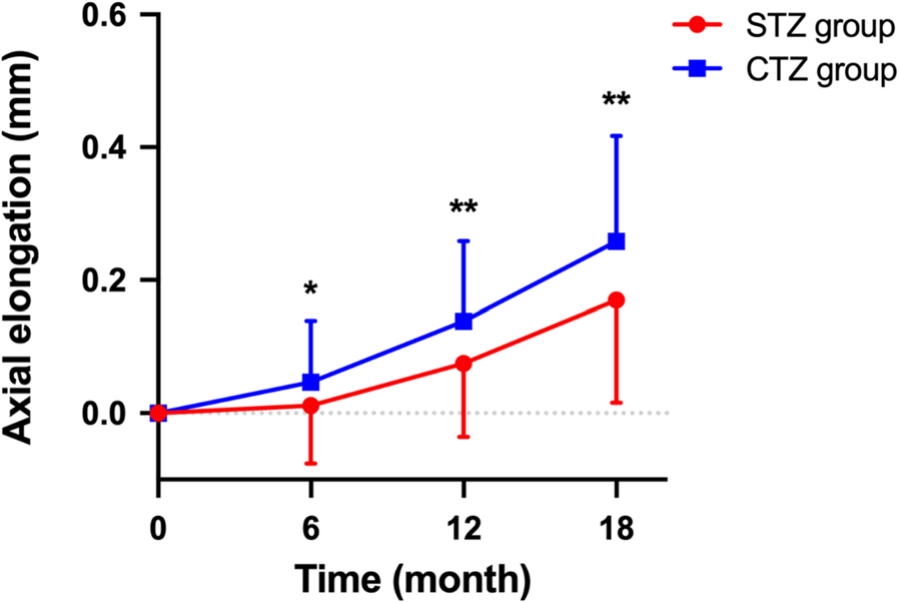
Axial elongation from baseline over 18 months visits in the two groups. Error bars represent the standard deviation. (STZ group, CTZ group: wearing orthokeratology lenses of small treatment zone vs. conventional treatment zone, respectively). STZ, small treatment zone; CTZ, conventional treatment zone; *P < 0.05; **P < 0.01

Age, sex, and SER could not confound interference with group and the changes in AL (*P*_age_ = 0.156; *P*_sex_ = 0.592; *P*_SER_ = 0.732). During the 18-month follow-up, a multifactorial repeated measures ANCOVA revealed significant effects of time (*F* = 21.111, *P* < 0.001), group (*F* = 9.775, *P* = 0.002) and group by time (*F* = 6.459, *P* = 0.007) on the axial elongation at different times. At the 6-, 12- and 18-month visits, the STZ group showed significantly smaller changes in AL compared to the CTZ group (Fig. 3; 6 months: 0.01 ± 0.09 mm vs. 0.05 ± 0.09 mm, *P* = 0.026; 12 months: 0.07 ± 0.11 mm vs. 0.14 ± 0.12 mm, *P* = 0.002; 18 months: 0.17 ± 0.15 mm vs. 0.26 ± 0.16 mm, *P* = 0.002). These results indicate that the STZ ortho-K lenses significantly slowed down AL elongation during the 18-month follow-up period, resulting in a reduction of 0.09 mm compared to the CTZ group (Table 2). At the 1-month visit, both groups showed significant improvements in SER and UCVA compared to baseline (*P* < 0.001), but there was no significant difference between the groups (*P*_SER_ = 0.913;* P*_UCVA_ = 0.332; Table 2).

**Table 2:** Changes in SER, UCVA, AL and topographic map parameters after orthokeratology treatment

Variable (mean ± SD)	STZ group (n = 68)	CTZ group (n = 63)	t value	*P* value
SER at 1 month (D)	0.04 ± 0.57	0.05 ± 0.45	− 0.110	0.913
UCVA at 1 month (logMAR)	0.00 ± 0.01	0.02 ± 0.12	− 0.974	0.332
AL changes at 6 months (mm)	0.01 ± 0.09	0.05 ± 0.09	− 2.266	0.025*
AL changes at 12 months (mm)	0.07 ± 0.11	0.14 ± 0.12	− 3.291	0.001*
AL changes at 18 months (mm)	0.17 ± 0.15	0.26 ± 0.16	− 3.407	0.001*
Changes in HOA (μm)	0.90 ± 0.43	0.71 ± 0.47	2.292	0.024*
Changes in total SA (μm)	0.37 ± 0.25	0.25 ± 0.29	2.477	0.015*
TZ diameter (mm)	2.50 ± 0.23	2.77 ± 0.18	− 7.529	< 0.001*
TZ area (mm^2^)	4.94 ± 0.89	6.05 ± 0.82	− 7.425	< 0.001*
Defocus ring width (mm)	2.45 ± 0.28	2.30 ± 0.30	2.814	0.006*
Total amount of defocus (D·mm^2^)	119.38 ± 63.71	91.40 ± 40.83	3.015	0.003*
Distance of decentration (mm)	0.41 ± 0.16	0.41 ± 0.16	0.096	0.923

Normally distributed data were expressed as mean ± standard deviation

*SER =* spherical equivalent refraction; *UCVA =* uncorrected visual acuity; *AL =* axial length; *HOA =* higher-order aberration; *SA =* spherical aberration; *TZ =* treatment zone; *Total amount of defocus =* the total value of defocus·area (D·mm^2^); *n =* number of subjects

^*^P < 0.05 indicates statistical significance

### Changes of topography parameters including defocus indicators and treatment zone size

After 1 month of treatment, the SA change in the STZ group was larger than that in the CTZ group (0.37 ± 0.25 μm vs. 0.25 ± 0.29 μm, *P* = 0.015). Analysis of tangential subtractive maps was performed by MATLAB ortho-K topography analysis software (specially written by this project team). The mean photopic PD of these children in both groups was 4.70 mm, so we took this PD value to limit the range for calculating the defocusing parameters and the diameter of the treatment area (Table 2). The corneal TZ diameter and TZ area in the STZ group were significantly smaller than those in the CTZ group (TZ diameter: 2.50 ± 0.23 mm vs. 2.77 ± 0.18 mm, *P* < 0.001; TZ area: 4.94 ± 0.89 mm^2^ vs. 6.05 ± 0.82 mm^2^,* P* < 0.001). The results showed evidence of a wider defocus ring within the pupil range (2.45 ± 0.28 mm vs. 2.30 ± 0.30 mm, *P* = 0.006) of the STZ group, as well as producing larger total amounts of defocus (119.38 ± 63.71 D·mm^2^ vs. 91.40 ± 40.83 D·mm^2^, *P* = 0.003). According to the definition of the decentration scale of the TZ [29], the decentration of the TZ was mild (mean eccentric distance < 0.5 mm) in both groups (0.41 ± 0.16 mm vs. 0.41 ± 0.16 mm, *P* = 0.923).

### Changes of parameters in objective visual quality

The masked intra-examiner ICC values (0.886) demonstrated high repeatability of OQAS-II measurements. The baseline OQAS parameters were similar between the two groups (all *P* > 0.05). After 1-month treatment, both groups showed a significant decrease in MTF cutoff and SR values compared to baseline (*P* < 0.001). The reduction in MTF cutoff value in the STZ group was about 1.3 times that of the CTZ group and about 1.5 times in the SR value (∆MTF cutoff: − 14.24 ± 10.48 vs. − 10.74 ± 9.46,* P*_MTF cutoff_ = 0.047; ∆SR: − 0.09 ± 0.07 vs. − 0.06 ± 0.07, *P*_SR_ = 0.026; Figure S2, Table 3). Additionally, MTF curves were plotted for both groups before and after 1-month of ortho-K treatment (Figure S3). It can be observed that MTF values decreased in both groups after treatment, with a more significant decrease in MTF values at the same spatial frequency in the STZ group. Pearson correlation analysis was conducted between changes of total SA and MTF cutoff variables, and a significant negative correlation was found (*r* =  − 0.202, *P* = 0.025). Following 1 month of wearing, both groups exhibited significantly higher OSI values compared to baseline (*P* < 0.001). Moreover, the STZ group showed a 1.4-fold elevation in the increment of OSI compared to the CTZ group (∆OSI: 0.84 ± 0.72 vs. 0.58 ± 0.53, *P*_OSI_ = 0.019).

**Table 3:** Comparison of objective visual quality parameters between the two groups in myopic children at Baseline and 1 month after orthokeratology treatment

Variable	MTF cutoff (c/deg)	SR	OSI	PVA100% (logMAR)	PVA20% (logMAR)	PVA9% (logMAR)
STZ (Baseline)	44.36 ± 8.22	0.27 ± 0.06	0.40 ± 0.22	− 0.16 ± 0.10	− 0.04 ± 0.11	0.15 ± 0.12
STZ (1 month)	30.43 ± 9.36	0.18 ± 0.05	1.23 ± 0.76	0.02 ± 0.15	0.16 ± 0.16	0.37 ± 0.15
∆a	− 14.24 ± 10.48	− 0.09 ± 0.07	0.84 ± 0.72	0.18 ± 0.15	0.20 ± 0.16	0.22 ± 0.14
CTZ (Baseline)	41.93 ± 8.55	0.24 ± 0.06	0.47 ± 0.26	− 0.13 ± 0.10	− 0.01 ± 0.12	0.19 ± 0.11
CTZ (1 month)	29.70 ± 10.90	0.19 ± 0.05	1.00 ± 0.59	0.03 ± 0.15	0.17 ± 0.16	0.35 ± 0.15
∆b	− 10.74 ± 9.46	− 0.06 ± 0.07	0.58 ± 0.53	0.15 ± 0.15	0.17 ± 0.16	0.16 ± 0.16
t1	1.653	1.891	− 1.818	− 1.460	− 1.375	− 1.793
*P*1	0.101	0.061	0.071	0.147	0.172	0.075
t2	9.217	8.677	− 8.701	− 8.037	− 8.309	− 9.618
*P*2	< 0.001*	< 0.001*	< 0.001*	< 0.001*	< 0.001*	< 0.001*
t3	7.012	5.739	− 6.512	− 7.218	− 7.411	− 6.914
*P*3	< 0.001*	< 0.001*	< 0.001*	< 0.001*	< 0.001*	< 0.001*
t4	− 2.002	− 2.253	2.369	0.834	0.869	2.085
*P*4	0.047*	0.026*	0.019*	0.406	0.386	0.039*

Normally distributed data were expressed as mean ± standard deviation

*STZ =* small treatment zone group; *CTZ =* conventional treatment zone group; *PVA =* predicted visual acuity; *MTF cutoff =* modulation transfer function cutoff frequency; *SR =* Strehl ratio; *OSI =* objective scatter index; *∆a =* STZ (1 month) − STZ (Baseline); ∆*b =* CTZ (1 month) − CTZ (Baseline)

*P*1: comparison of each parameter between two groups before orthokeratology treatment; *P*2: comparison of the STZ group before and after orthokeratology treatment; *P*3: comparison of the CTZ group before and after orthokeratology treatment; *P*4: comparison of ∆a and ∆b; (STZ group: n = 68, CTZ group: n = 63, n: number of subjects)

^*^P < 0.05 indicates statistical significance

After 1 month of treatment, both groups exhibited significant decreases in PVA100%, PVA20%, and PVA9% compared to baseline (all *P* < 0.001). The reduction in PVA9% demonstrated a significant difference between the two groups (∆PVA9%: 0.22 ± 0.14 vs. 0.16 ± 0.16, *P* = 0.039), while no statistical difference was observed in the reductions of PVA100% and PVA20% (∆PVA100%: 0.18 ± 0.15 vs. 0.15 ± 0.15,* P* = 0.406; ∆PVA20%: 0.20 ± 0.16 vs. 0.17 ± 0.16, *P* = 0.386).

### Univariate and multivariate linear regression

To analyze the factors affecting the 18-month AL elongation, regression analyses included parameters of age, sex, SER, PD, total amount of defocus, defocus ring width, distance of decentration, changes of MTF cutoff value, total SA, and TZ diameter. In Table S2, the univariate linear regression analysis showed significant correlations with the above factors (all *P* < 0.001). In the multivariate linear regression model, AL changes were significantly associated with sex (beta = − 0.075, *P* = 0.012), the change of MTF cutoff value (beta = 0.004, *P* = 0.010), the increment of total SA (beta = 0.170, *P* = 0.003), and the TZ area (beta = 0.044, *P* = 0.001). The multivariate regression model had a good model fit (*r*^*2*^ = 0.710, *P* < 0.001).

### Subjective visual quality questionnaire results

All participants completed the subjective visual quality questionnaire and their results showed that there was no significant difference between the two groups (27.78 ± 0.91 vs. 27.89 ± 0.80, *P* = 0.666), and none of the scores of individual subjective parameters in the questionnaire were statistically significant (Table S3). This suggests that there is no difference in subjective visual quality between children wearing different TZ ortho-K lenses.

### Safety

Adverse events were observed in two myopic children of the CTZ group during the 18-month follow-up visit, with one subject (2%) reporting allergic conjunctivitis at the first-month visit, which subsided with treatment after discontinuing ortho-K lens wear. One subject (2%) had recurrent corneal staining during the fourth month of wear and therefore stopped wearing the lens, recovered from treatment and withdrew from the study in favor of another ortho-K lens. In addition, four subjects (6%) in the CTZ group were dissatisfied with the control of myopia progression and withdrew from the study.

## Discussion

In tandem with results from our study, others have found that conventional VST-designed ortho-K lenses can slow the progression of myopia in children [30]. However, the ortho-K lenses with the STZ design could better retard AL elongation during the 18-month treatment period, while the quality of retinal visual signals decreased moderately after treatment when compared with CTZ lenses. Regarding the characteristic of post-therapeutic corneal topography for STZ lenses, it showed a smaller TZ diameter, a wider defocus ring width, and a larger value of effective total amount of defocus within PD range and increased total SA. Analyses suggest that these characteristics—smaller anterior corneal treatment zone, increased SA due to corneal morphology changes, and decreased contrast intensity of visual signals on the retina—may contribute to the efficacy of this new ortho-K lens design in slowing myopia progression.

Previous reports have highlighted the design of ortho-K lens in reducing the BOZD to achieve a smaller TZ on the corneal anterior surface, which is different from our lens modification. Lin et al. found that a smaller diameter of the effective TZ corresponds to better control of AL [18]. Pauné et al. compared the effectiveness of myopia control in Caucasian children wearing ortho-K lenses with different BOZD and reported that a BOZD of ≤ 5.0 mm resulted in a reduced TZ and a better retardation of AL elongation [31]. Guo et al. showed that in 2-year clinical trial, there was better control of AL in children wearing ortho-K lenses with a 5-mm BOZD (0.15 ± 0.21 mm) compared with those wearing lenses with a 6-mm BOZD (0.35 ± 0.23 mm) [19]. Studies have also shown that the efficacy of ortho-K lenses decreases with time of wearing [8]. The study by Guo’s group showed that the improvement in myopia from lenses with the smaller BOZD occurred mainly in the first six months of treatment [20], and our results are shown to be consistent with this finding, strongly suggesting that the efficacy at the early stages was more pronounced than in the later stages. In our study, the axial elongation of the STZ group was 0.07 mm at 12 months and 0.17 mm at 18 months. Compared with the CTZ group, the axial elongation in the STZ group was reduced by 0.07 mm and 0.09 mm, respectively. According to a previous study, there is a relationship between changes in spherical equivalent refraction and alterations in AL [32]. This relationship can be quantified as a refractive error change of approximately 0.16 D and 0.20 D, respectively, which is considered a significant clinical change.

The Pentacam systematically underestimates pupil size while the IOL-Master measures PD in children with a high degree of reproducibility [33, 34], so we obtained PD values by the IOL-Master instead of the Pentacam. The mean value of 4.70 mm was taken as PD and used to limit the analyzing range in the subtractive map to obtain an effective corneal defocus shift within the PD range. Our results suggest that the STZ ortho-K lens produced a significantly smaller TZ (2.50 ± 0.23 mm vs. 2.77 ± 0.18 mm, *P* < 0.001), a wider defocus ring (2.45 ± 0.28 mm vs. 2.30 ± 0.30 mm, *P* = 0.006) on the cornea surface, and more effective corneal defocus (119.38 ± 63.71 D·mm^2^ vs. 91.40 ± 40.83 D·mm^2^, *P* = 0.003) within the PD, leading to better myopic control. Previous studies used another corneal refractive parameter to describe the morphological change of the cornea to predict the effect of myopia control [35]. By observing the relative corneal refractive power (RCRP) shift, myopia degrees induced by the ortho-K lens on the peripheral retina can be inferred. Yang et al. proposed that the RCRP in the central region was more effective in preventing myopia development than that in a peripheral region [36]. Li et al. proposed that an ortho-K lens with a smaller BOZD design resulted in a steeper RCRP profile distribution within the PD [37].

A known challenge of wearing ortho-K lenses is the potential compromise of visual quality, including reduced contrast sensitivity, decreased MTF values and increased aberrations [14], making it a worse experience for myopic children wearing STZ lenses. Our previous study observed significant decreases in MTF cutoff and increases in OSI values after ortho-K lens wearing [15]. Carracedo et al. found that contrast sensitivity significantly decreased when wearing 5 mm TZ lenses, but not when wearing 6 mm TZ lenses [38]. The OQAS results of this current study showed a much more obvious reduction in MTF cutoff (− 14.24 ± 10.48 vs. − 10.74 ± 9.46, *P* = 0.047), SR (− 0.09 ± 0.07 vs. − 0.06 ± 0.07, *P* = 0.026) and PVA values as well as a significant increase in OSI (0.84 ± 0.72 vs. 0.58 ± 0.53, *P* = 0.019) in the STZ group. Before the commencement of the study, we performed a basic examination of the child’s eye condition to exclude intraocular diseases. Therefore, we hypothesized that the elevated scatter caused by ortho-K treatment, mainly came from uneven corneal refraction within the optical zone. In addition, we found that only the decrement of PVA9% showed a significant difference between the two groups, indicating that there was no difference in visual acuity under normal or slightly poorer light conditions, with changes occurring only under low light conditions such as at nighttime. Furthermore, the subjective visual quality questionnaire scores did not show a significant difference between the two groups, indicating that the STZ ortho-K lenses did not impact on subjective visual quality of the myopic children.

Recent studies have found that the visual quality of images formed on the retina i.e., the contrast of visual signals on the retina, not only affected the clarity of the subject’s vision but also influenced axial elongation, which impacts the progression of myopia. In vivo experiments indicate that retinal contrast signal and defocus may affect the occurrence of eye refraction [39]. Ortho-K treatment has been shown to slow the progression of myopia mainly due to myopia defocus formed at the peripheral retina [40, 41]. However, others believe that the reduction of contrast intensity of the light signal is an accompanying factor [42]. In addition to MTF cutoff values, aberrations were also considered a factor for visual quality. Significant increases in HOAs and coma aberrations were reported after wearing ortho-K lenses, which exhibited a negative correlation with axial elongation [43]. In this study, the increment of total SA was larger in the STZ group (0.37 ± 0.25 μm vs. 0.25 ± 0.29 μm, *P* = 0.015) after 1 month of lens wearing. We chose to correlate the change of total SA with the MTF cutoff value, and a negative correlation was found between the two indicators (*r* =  − 0.202, *P* = 0.025). STZ ortho-K lenses reshaped the cornea and resulted in a smaller TZ on the corneal surface, producing more SA and reducing contrast intensity of the visual signal on the retina, then confused the interpretation of retinal signals and led to a slower axial elongation. This finding was consistent with a previous study that evaluated the variation of orthokeratology lens treatment zone (VOLTZ). The authors proposed that small BOZD lenses produced a smaller TZ, resulting in a larger change in HOAs at the corneal surface, which improves myopia control [44].

The effectiveness of ortho-K lenses in controlling myopia progression may be associated with several factors. Older age, higher baseline refractive error, larger PD, higher aberrations, greater myopic defocus, and significant decentration have been identified as factors associated with slower AL growth after ortho-K treatment [18, 45–47]. In our study, the multiple linear regression model was constructed with a goodness of fit (*R*^2^ = 0.710), indicating that AL elongation was determined by factors such as the TZ area, SA, MTF cutoff value and sex. As for sex, we speculate that this may be due to the effect of the preponderance of girls among the myopic children in both groups. It has also been shown that at a given age, girls are twice as likely to be myopic as boys [48]. Studies have also shown that ortho-K lenses with smaller TZ can provide more effective control of axial elongation by generating greater peripheral retinal defocus near the fovea [38]. Our findings show that STZ ortho-K lenses produce a smaller TZ with a wider defocus ring, and thus more defocus enters the range of the pupil. This also leads to an increase in SA, which causes a deterioration in visual quality thereby confusing the retina's analysis of visual signals to prevent axial elongation, and ultimately achieve better myopia control. Hence, this improvement in myopia control may come at the expense of poorer visual quality.

## Limitations

First, we chose to observe changes in objective visual quality parameters at the 1-month follow-up. This is since the remodeling effect of ortho-K on the eye reaches a stable level within 1 month [49–51]. Moreover, other studies have shown no significant difference in objective visual quality parameters between the 1-month and 3-month follow-ups [52]. Secondly, this study lacked a control group of myopic children who received no treatment or wore single-vision spectacles. Since single-vision spectacles do not slow down axial elongation in children [30], recruiting untreated myopic children or those wearing single-vision spectacles would be unethical. Moreover, if children were randomly assigned to the control group, they might drop out due to the rapid progression of myopia. Finally, our trial included only myopic children of Chinese descent and did not account for other ethnic groups. Therefore, the results may not be generalizable to children of other ethnic backgrounds. Future studies would include diverse ethnic groups to validate the findings.

## Conclusions

The STZ ortho-K lens can be achieved by different lens designs. In this study, a smaller treatment zone on the anterior surface of the cornea was created by varying the depth of the reversed zone. Compared to conventional ortho-K lenses, the axial elongation in myopic children becomes slower when wearing STZ ortho-K lenses. However, at the same time, there was a certain reduction in the quality of vision. Smaller TZ on the corneal surface formed after STZ ortho-K wearing, which then produced a greater SA, resulting in the lower contrast intensity of retina visual signal. This, in turn, led to a better control of myopia progression. Axial elongation was affected by retinal visual quality, and it may be a mechanism by which ortho-K prevents myopia progression.

### Supplementary Information

Additional file 1.

## Data Availability

The datasets generated/analyzed during the current study are available from the corresponding author upon reasonable request.

## Abbreviations

AL: Axial length
ANCOVA: Analysis of covariance
BC: Base curve
HOA: Higher-order aberration
IOP: Intraocular pressure
MTF: Modulation transfer function
Ortho-K: Orthokeratology
OQAS-II: Optical quality analysis system-II
OSI: Objective scatter index
PD: Pupil diameter
PVA: Predicted visual acuity
PSF: Point spread function
RCRP: Relative corneal refractive power
RZD: Reverse zone depth
STZ: Small treatment zone
SA: Spherical aberration
SER: Spherical equivalent refraction
SR: Strehl ratio
TZ: Treatment zone
UCVA: Uncorrected visual acuity
VOLTZ: Variation of orthokeratology lens treatment zone
