# Supplementary material for: Efficacy of orthokeratology lens with the modified small treatment zone on myopia progression and visual quality: a randomized clinical trial
Source: Eye Vis (Lond). 2024 Sep 2;11:35. doi: 10.1186/s40662-024-00403-3 (PMC11367740; doi:10.1186/s40662-024-00403-3)

**Supplementary Information**

**Table S1.** The parameters of different design of orthokeratology lenses.

| **Parameters** | **STZ OK lens** | **CTZ OK lens** |
| --- | --- | --- |
| Brand | CRT; Paragon Vision Sciences, USA | VST; Alpha Corp., Japan |
| Material | HDS 100 | BOSTON EM |
| Oxygen permeability (cm^2^/s[mLO_2_/(mL$\times$mmHg]) | 100 | 104 |
| Lens design | Modified three-zone design with an elevated depth of the reverse zone | Conventional four-zone |
| Back surface geometry | Sigmoid geometry | Reverse geometry |
| Back optical zone diameter (mm) | 6.0 | 6.0 |
| The widths of the other zones (mm) | LZ: 1.5/ RZ: 1.0/ OZ: 6.0 | BC: 6.0/ RC: 0.6/ AC: 1.3/ PC: 11.0 |
| Lens diameter (mm) | 10.0/10.5/11.0 | 10.0–11.0 |
| Central thickness (mm) | 0.16 | 0.22 |

OK = orthokeratology; LZ = landing zone; RZ = reverse zone; OZ = optical zone; BC = base curve; RC = reverse curve; AC = alignment curve; PC = peripheral curve

**Table S2.** Univariable and multivariable analysis of the associations between potential factors and changes in 18-month AL.

| **Parameters** | **Univariable analysis** | | | **Multivariable analysis** | |
| --- | --- | --- | --- | --- | --- |
|  | **Beta (95% CI)** | ***P*** | | **Beta (95% CI)** | ***P*** |
| Age (years) | 0.020 (0.017 to 0.023) | | < 0.001***** | −0.012 (−0.028 to −0.005) | 0.163 |
| Sex (M/F) | 0.115 (0.097 to 0.133) | | < 0.001***** | −0.075 (−0.134 to −0.017) | **0.012*** |
| SER (D) | −0.081 (−0.094 to −0.067) | | < 0.001***** | −0.001 (−0.032 to 0.030) | 0.962 |
| PD (mm) | 0.043 (0.037 to 0.049) | | < 0.001***** | 0.010 (−0.019 to 0.038) | 0.503 |
| Total amount of defocus (D·mm^2^) | 0.001 (0.001 to 0.002) | | < 0.001***** | 0.000 (−0.001 to 0.000) | 0.190 |
| Defocus ring width (mm) | 0.089 (0.077 to 0.101) | | < 0.001***** | 0.076 (−0.006 to 0.158) | 0.068 |
| Changes in MTF cutoff (c/deg) | −0.009 (−0.012 to −0.007) | | < 0.001***** | 0.004 (0.001 to 0.007) | **0.010*** |
| Changes in total SA (μm) | 0.334 (0.279 to 0.388) | | < 0.001***** | 0.170 (0.058 to 0.281) | **0.003*** |
| Decentration (mm) | 0.451 (0.381 to 0.522) | | < 0.001***** | −0.014 (−0.205 to 0.177) | 0.883 |
| TZ area (mm^2^) | 0.038 (0.033 to 0.043) | | < 0.001***** | 0.044 (0.019 to 0.070) | **0.001*** |
| Final model | R^2^=0.710 | | *P*< 0.001***** |  |  |

SER = spherical equivalent refraction; PD = pupil diameter; MTF cutoff = modulation transfer function cutoff; SA = spherical aberration; TZ = treatment zone. **P* < 0.05 indicates statistical significance.

**Table S3.** Values of the subjective visual quality questionnaire.

| **Parameters** | **STZ group**  **(n=68)** | **CTZ group**  **(n=63)** | **t value** | ***P* value** |
| --- | --- | --- | --- | --- |
| Near vision | 4.79 ± 0.44 | 4.71 ± 0.26 | 1.225 | 0.184 |
| Far vision | 4.44 ± 0.25 | 4.49 ± 0.10 | −1.719 | 0.089 |
| Vision stability at near | 4.88 ± 0.32 | 4.89 ± 0.33 | −0.298 | 0.767 |
| Vision stability at distance | 4.88 ± 0.40 | 4.87 ± 0.38 | 0.081 | 0.935 |
| Glare | 4.44 ± 0.33 | 4.44 ± 0.40 | −0.074 | 0.941 |
| Satisfaction with correction | 4.36 ± 0.37 | 4.44 ± 0.27 | −1.360 | 0.176 |
| Overall score | 27.78 ± 0.91 | 27.89 ± 0.80 | −0.432 | 0.666 |

STZ group = small treatment zone group; CTZ group = conventional treatment zone group; Normally distributed data were expressed as mean ± standard deviation. **P* < 0.05 indicates statistical significance.

**Figure S1.** Comparison of corneal topography between the STZ group and the CTZ group. **a** Corneal topographic map of a patient in the small treatment zone (STZ) group; **b** Corneal topographic map of a patient in the conventional treatment zone (CTZ) group.


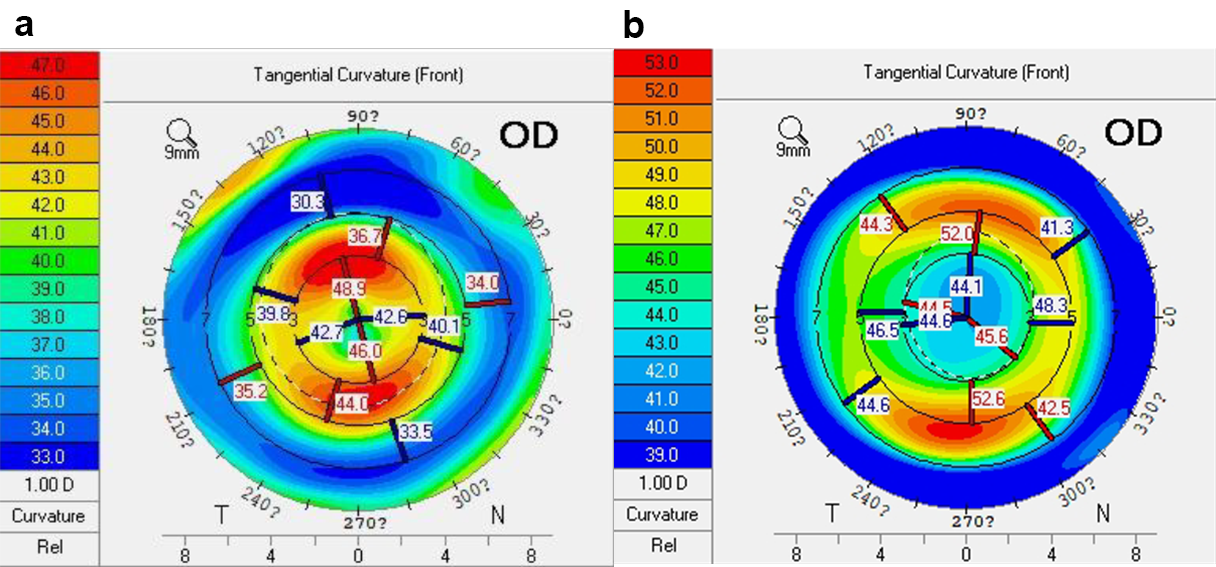


**Figure S2.** Comparison of OQAS parameters at baseline and 1-month of orthokeratology treatment in two groups. **a** Modulation transfer function (MTF) cutoff; **b** Strehl ratio; **c** Objective scatter index. STZ, small treatment zone; CTZ, conventional treatment zone


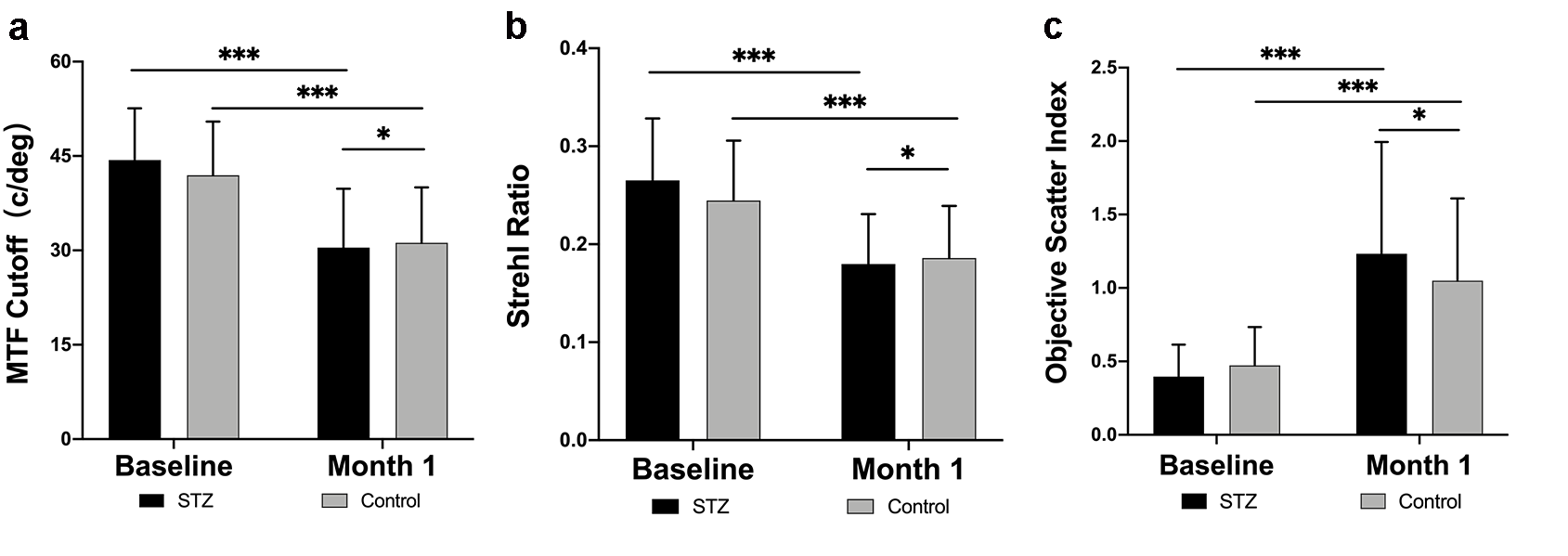


**Figure S3.** Changes in modulation transfer function at baseline and 1-month of orthokeratology treatment between two groups. MTF, modulation transfer function; STZ, small treatment zone; CTZ, conventional treatment zone


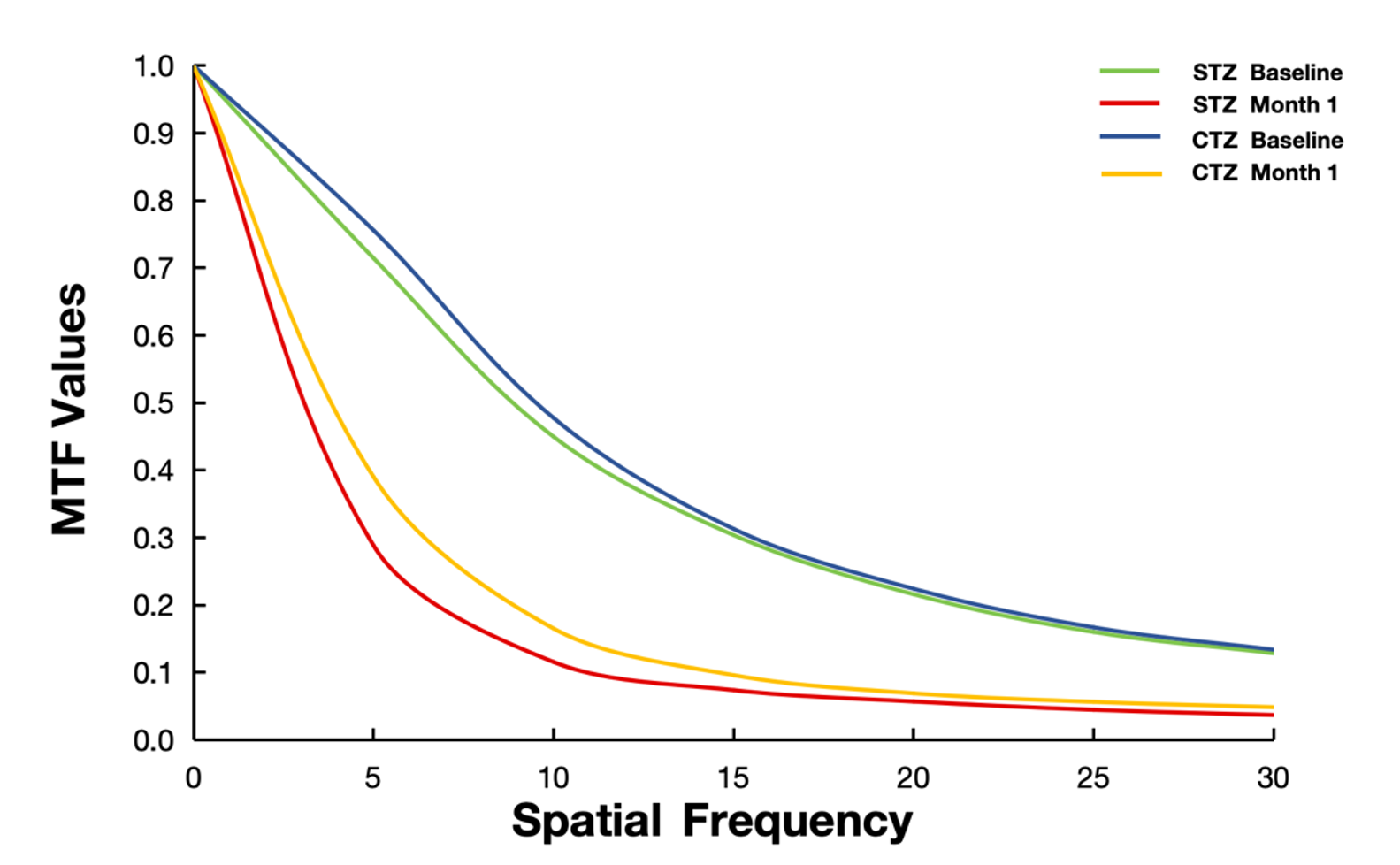

Supplement: Supplementary file 1 — Additional file 1. [file 40662_2024_403_MOESM1_ESM.docx]
